# Efficacy in the use of gamification strategy in phonological therapy

**DOI:** 10.1590/2317-1782/20232022181en

**Published:** 2023-09-08

**Authors:** Thalia Freitas da Silva, Grazielly Carolyne Fabbro Ribeiro, Cássio Eduardo Esperandino da Silva, Mayara Ferreira de Assis, Henrique Dezani, Larissa Cristina Berti

**Affiliations:** 1 Departamento Fonoaudiologia, Faculdade de Filosofia e Ciências, Universidade Estadual Paulista “Júlio de Mesquita Filho” - UNESP - Marília (SP), Brasil.; 2 Departamento Ciência da Computação, Faculdade de Tecnologia - FATEC - São José do Rio Preto (SP), Brasil.

**Keywords:** Phonological Disorder, Speech Therapy, Speech Perception, Gamification, Therapy Computer Assisted, Games Experimental

## Abstract

**Purpose:**

to compare the efficacies of traditional phonological therapy and phonology associated with the gamification strategy in children with Phonological Disorder (PD).

**Methods:**

ten individuals with PD participated who showed the process of replacing liquids. They were randomized into two groups: traditional phonological therapy (control group - CG) and phonological therapy associated with a gamification strategy mediated by computer (gamification group - GG). The phonological intervention comprised, for both groups, stages of speech perception and production. Interventions differed in the perception stage, in which the GG was submitted to the game with gamification strategies. At the end of each session, individuals speech production (% of correct answers) were registered for each therapeutic stage, based on target words and sounding words. For analysis the following were considered: The individuals mean of correct answers for each therapeutic stage; PCC-R value (percentage of correct consonants) pre and post therapy; beyond of the number of sessions used to reach 85% of correct production.

**Results:**

there was no statistical difference between the types of intervention considering the average of correct answers of the productions and the number of sessions. There was a significant effect for pre- and post-therapy conditions in the comparison PCC-R values ​​for both models. The individuals in the GC had the PCC-R values higher than those of GG.

**Conclusion:**

both models of intervention present similar results, providing an improvement in the individuals phonological performance from the first session.

## INTRODUCTION

Gamification refers to using game mechanisms [e.g., toy, play or ludicity (analog or digital)] to awaken engagement among a specific audience. These strategies make up game-design resources and game elements in non-game contexts, with their differential being the ability to establish rewards, scoring, challenges, reinforcement, feedback and ranking^(1-2)^.

Among the areas of knowledge that can benefit from strategies associated with gamification is Speech Therapy, which can be employed in the evaluation and/or intervention stages. In recent years, gamification strategies have emerged in all areas of Speech Therapy, and their use has gradually increased, associated or not with games. Consequently, a product of this resource is the proposition and sale of various software involving games for assessing and treating subjects with different communication disorders. For example, Pedro’s Spooky Night (Pedro em uma Noite Assustadora) stimulates phonological and phonoarticulatory awareness. Additionally, the Phonological Assessment Instrument (Instrumento de Avaliação Fonológica - INFONO) facilitates the assessment process of subjects with speech disorders, and the Hearing Disorders Rehabilitation Aid (Auxiliar na Reabilitação de Distúrbios Auditivos - SARDA) stimulates cognitive-auditory-visual processing skills^(3-6)^.

Notably, among the potential users of these materials, some subjects present the diagnosis of Speech Sound Disorder, specifically Phonological Disorder (PD). Subjects with PD presented unexpected speech production for their age and development stage. In other words, they continue to use simplification rules (i.e., phonological processes) beyond the expected age without any apparent organic etiology^(7)^.

Over the years, studies that include phonological intervention have intensified to ascertain its efficacy and efficiency in the adult Brazilian Portuguese population. The intervention process in individuals with PD primarily aims to reorganize the altered phonological system and improve speech intelligibility^(8-11)^. However, for this to happen, the subjects need to be motivated to carry out the activities proposed in the therapy that involves the ability in which they have the greatest difficulty, speech production.

In this sense, it becomes a daily challenge for clinical speech therapists to develop strategies that motivate individuals with PD to help them work on their production difficulties, considering that, in general, the interest of children is focused on different technologies, like computers and other electronic devices^(12)^. Thus, computer-based gamification strategies in the context of phonological therapy could be essential and/or complementary to traditional therapy methods.

In Brazil, a study using the software FonoSpeak^(12)^, which aids in the acquisition and training of phonemes and activities elaborated in the Microsoft Office PowerPoint program, the authors verified that the therapy group submitted to computer-based gamification strategies obtained a more significant number of correct productions when compared to the group of subjects submitted to traditional therapy, without gamification. 

In agreement with the study mentioned above, a research^(13)^ aiming to evaluate the effectiveness of therapeutic programs with the use of computer in subjects with Speech Sound Disorders, including PD, from the literature review of 14 studies, found that although the evidence based on computer use is still gaining ground, the literature has shown that this resource can be a valuable adjunct to therapy.

However, there is contradictory evidence in the literature about the benefit of using gamification strategies with computer resources in treating individuals with PD^(14)^. This study compared the performance of PD subjects using three groups: subjects submitted to a gamification strategy therapy (experimental software with interactive computer games), traditional therapy and no therapy. No significant difference existed between the groups based on analyzing the subjects' speech production performance.

Similarly, in another study^(15)^, the effectiveness of the traditional phonological intervention was compared with the phonological intervention based on the use of the tablet, therefore, the subjects were randomized for each group. The authors concluded that there was no statistical difference between the groups, which means that regardless of the group, the subjects obtained percentages of correct speech production in the post-therapy condition.

There is also in the national literature, a research^(16)^ different from those mentioned above, which sought to describe the frequency of the tablet tool during intervention of subjects with PD. The gamification games used on the tablet were not of a speech-language nature but adapted to work on imitation/naming tasks at the levels of syllables, words and phrases. The tablet was used as an auxiliary route; therefore, it was available for the subject to request or not use during therapy. The study included four subjects aged between five years and five months to five years and eleven months. All subjects requested the tablet with significant frequency during the sessions, functioning as a motivating resource; however, not decisive for the evolution of the cases.

The studies, taken together, show not only contradictory results about the advantage of using gamification strategies in phonological therapy but also offer little scientific evidence insofar as the sampling was not random or the method used did not allow the comparison between proposals of different therapies.

Assuming that the use of gamification strategies in the therapeutic process of subjects with PD could favor their engagement in activities and, consequently, favor the learning of the speech production skill worked on in therapy, it was hypothesized that subjects who receive phonological intervention associated with gamification strategies would present a better performance in terms of percentage of correct answers for the worked skill and a shorter therapeutic time when compared to subjects submitted to traditional phonological therapy. Therefore, the present study aimed to compare the effectiveness of phonological therapy associated with the gamification strategy with the efficacy of traditional therapy in subjects with PD.

## METHODS

This study was prospective and longitudinal. It was approved by the Research Ethics Committee of the Faculty of Philosophy and Sciences/São Paulo State University (FFC/UNESP), Campus Marília, under protocol nº 4.615.118.

The subjects' parents and/or guardians signed the Informed Consent Form, authorizing their participation in the project and the publication of the results. At the beginning of each therapeutic intervention session, the participating subjects were asked if they would agree to participate. All children expressed their respective acceptance.

The recruited sample totaled 86 subjects and was based on speech-language screening, which included speech and language assessment, on Basic Health Unit and the Specialized Center in Rehabilitation. Both sites are located in the interior of São Paulo state, in the city of Marília. Therefore, the sampling process of the present study was for convenience.

The research inclusion criteria required that the children were between four and eight years of age, diagnosed with PD, with the presence of the process of substituting a non-lateral liquid for a lateral one or vice versa (/ɾ/ 🡪 [l] or /l/ 🡪 [ɾ]), regardless of the severity of the PD or other associated phonological processes, and that the parents and/or guardians demonstrated interest and availability to participate in the proposed therapeutic intervention program.

Exclusion criteria included significant structural alterations of the phonoarticulatory organs; the presence of comorbidities in addition to the speech production complaint; complaints of hearing and/or hearing disorders, and non-adherence to the proposed intervention program or possible dropouts.

Ultimately, ten subjects participated in the study, six males and four females, aged between four years and eleven months to seven years and three months old, with a diagnosis of PD and who presented the phonological process of substituting non-lateral liquid for sideways or vice versa.

All subjects were submitted to a phonological assessment with the “Speech Assessment Instrument for Acoustic Analysis” (Instrumento de Avaliação de Fala para Análise Acústica - IAFAC) to establish the PD diagnosis and characterize the phonological processes^(17)^. This instrument consists of 96 words that include all consonant phonemes in Brazilian Portuguese (BP) in the context of /i to u/ in accented positions. For this evaluation, 28 words were used, in which all BP phonemes occur only in the context of the vowel /a/.

Furthermore, the PD severity index was calculated using the Percent Consonants Correct - Revised (PCC-R), which refers to the percentage of consonants produced correctly in relation to the percentage of the total number of consonants contained in the obtained speech sample. For the calculation of this index, only substituted or omitted phonemes are considered errors^(18)^.

The following criteria were considered: a) mild: above 85% of correct answers; b) slightly moderate: between 65% and 85% of correct answers; c) moderately severe: between 50% and 65% of correct answers; d) severe: below 50% of correct answers to classify the different degrees of PD^(19)^.

The subjects were also submitted to an audiological assessment to investigate auditory thresholds to rule out possible alterations.

After the characterization of the subjects, they were randomly selected and randomized into two groups: traditional phonological therapy (control group - CG) and phonological therapy associated with a computer-mediated gamification strategy (gamification group - GG). Both groups (CG and GG) had five subjects each. The subject profiles are presented in Table 1.

**Table 1:** Pre-therapeutic intervention subject profiles

Subject	Sex	Age	Phonological process - Liquid substitution	PCC-R Pre-intervention	PD Severity	Group
S1	M	6y 5m	L for N-L	89.7%	Mild	CG
S2	M	5y 11m	N-L for L	87.1%	Mild	GG
S3	F	4 y11m	N-L for L	77.6%	Mild-Moderate	GG
S4	F	7y 3m	N-L for L	91.5%	Mild	CG
S5	M	5y 11m	N-L for L	92.2%	Mild	CG
S6	M	5y 5m	N-L for L	94.1%	Mild	CG
S7	F	5y 5m	N-L for L	62.9%	Mild-Moderate	GG
S8	M	5y 0m	N-L for L	83.5%	Mild-Moderate	GG
S9	M	6y11m	N-L for L	57.3%	Moderate-Severe	GG
S10	F	6y 2m	N-L for L	82.9%	Mild-Moderate	CG

**Caption:** M = Male; F = Female; L for N-L = Substitution of Lateral Liquid for Non-Lateral; N-L for L = Replacement of Non-lateral Liquid for Lateral; GG = Gamification Group; CG = Control Group

In the phonological-based intervention program for the CG and GG groups, there were 16 individual speech therapy sessions, with a frequency of two weekly 50-minute sessions, during the subjects' after-school hours.

Thirty target words were selected with the phonemes /l/ and /ɾ/ in ISDP criteria (beginning of a syllable and within a word), worked on during the intervention stages, and another 30 probe words that were not worked on in therapy that served to observe the generalizations made by the subjects were used to conduct the intervention. As illustrated in Chart 1, part of the selected words refers to minimal pairs, that is, words that differ in only one phoneme.

**Chart 1:** Words used during the therapeutic intervention process in groups CG and GG

**TARGET WORDS**		**PROBE WORDS**	
**/ɾ/**	**/l/**	**/ɾ/**	**/l/**
VERA*	VELA/ CANDLE	VARETA/ ROD	VALETA/ DITCH
MARA*	MALA/ SUITCASE	CARANGO/ SNAP	CALANGO/ LIZARD
CARO/ DEAR	CALO/ CALLUS	BARÃO/ BARON	BALÃO/ BALLOON
PURO/ PURE	PULO/ JUMP	PÁRIO*	PÁLIO/ CANOPY
CARA/ FACE	CALA/ SHEET	CARUSO*	CALUSO*
SARA*	SALA/ LIVING ROOM	PERU/ TURKEY	PELU*
VIRA/ TURN	VILA/ VILLAGE	GARI*	GALI*
VARA/STICK	VALA*	PORO/ PORE	PÓLO/ POLE
CORADO/ FLUSHED	COLADO/ GLUED	RARA/ RARE	RALA*
CERA/ WAX	SELA/ SADDLE	GARO*	GALO/ ROOSTER
MORA*	MOLA/ SPRING	GERA/ GENERATE	PALA/ PAL
MARINHA/ NAVY	MALINHA/ SUITCASE	FARINHA/ FLOUR	BALA/ BULLET
MIRA/SIGHT	MILA*	JARARACA/ PIT VIPER	COLA/ GLUE
SARADA/ HEALED	SALADA/ SALAD	MARACÁ/ MARACA	BELA/ LOVELY
CORAGEM/COURAGE	COLAGEM/ COLLAGE	PERA/PEAR	PISTOLA/ PISTOL

**Caption:** (*) no possibility of translation to English

The 16 speech therapy sessions for the CG and GG consisted of speech perception and production stages, namely: 1) pre-intervention: initial collection/presentation and contextualization of the pictures corresponding to the selected words; 2) explanation of the phonological process, based on the contrastive value of the target words, it was explained to the subject, through a prototype of phonoarticulatory organs, that there are sounds that the language hits slowly as is the case of /l/ and there are sounds that the language hits fast as in the production of /ɾ/; 3) perception in the other, the subject performed auditory-visual perception immediately after speech production of the target sounds of the other (therapist); 4) perception itself, based on the production of the target sounds, the subject simultaneously performed auditory-visual proprioception. In this skill, the correct production of the target sound was not required; the subject should only perform his proprioception; 5) production: the subject should correctly produce the target sound (based on facilitating cues) in an isolated word and the construction of sentences; and 6) post-intervention: final collection.

The interventions differed in stage 3, related to the perception of the other, in which the CG relied exclusively on ludic activities with physical material, while the GG was submitted to the gamification game “Ho-ho roubaram as palavras” (English “Ho-ho stole the words”) mediated by the computer. Therefore, this group's subjects should perceive the target sounds from the speech of the other through the game.

**Figure 1:**
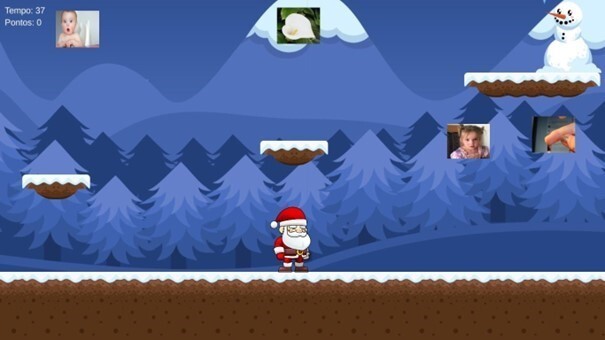
Interface of *“*Ho-ho stole the words”

**Source:** Unity WebGL Player | Papai Noel Game^(20)^

The game was developed in partnership with a team of professionals from Faculty of Technology (FATEC) of São José do Rio Preto in the interior of São Paulo state coordinated by Dr. Henrique Dezani. The developed platform can be used on computers, available online and free of charge. “Ho-ho roubaram as palavras”/”Ho-ho stole the words” has as its main character Santa Claus. The game aims to engage the subject to find the 30 target words hidden in the game scenario. As the subject finds the target words, the auditory stimuli corresponding to each figure, produced by the other, are presented so that the subject can identify the target sounds. It can be accessed via the link found in the references^(20).^ The game interface is shown in Figure 1.

### Data analysis

For both interventions, each subject's performance was recorded through speech production recordings, calculated from the percentage of correct answers for each therapeutic skill worked out of the 30 target and 30 probe words. The recordings of the subjects' speech productions were made only at the end of each session for subsequent auditory-perceptual analyses.

The recordings of speech productions were analyzed by at least two referees who performed the judgment based on three criteria: (A) target sound production success, (E) target sound production error or (G) gradient production. A total of 960 recordings were made for each subject. A third referee was necessary to confirm the analysis of 281 (118 recordings for target words and 163 recordings for probe words) recordings of all subjects in the sample.

For each therapeutic stage (perception in the other, perception in itself and production), the averages of correct answers were considered in terms of percentage for each CG and GG subject.

PCC-R values were compared in pre- and post-therapy conditions in both groups. An analysis was also made of the number of sessions required to reach 85% correct production between the types of intervention. The 16 proposed sessions were carried out regardless of whether they reached the established correct production.

Descriptive and inferential statistical analyzes were performed using the STATISTICA software (version 7.0). Repeated Measures ANOVA was utilized to compare group performance (CG vs. GG) in the therapeutic intervention stages and the PCC-R pre- and post-therapy values. The Post hoc test used was the Scheffé test. One-way ANOVA was used to compare the number of sessions in the two interventions. A value of α>0.05 was established.

## RESULTS

**Figure 2:**
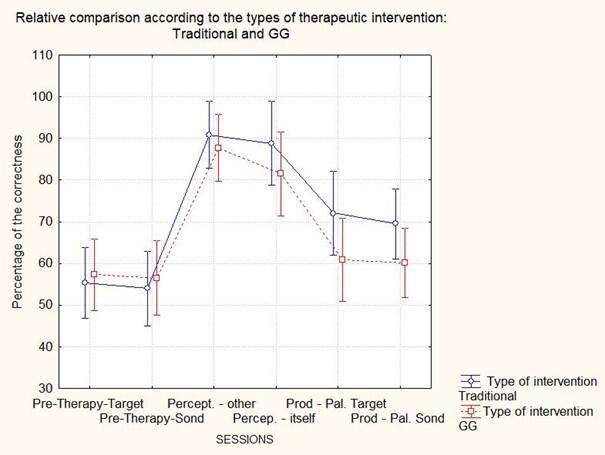
Relative comparison according to the types of therapeutic intervention: Traditional and GG

Table 2 presents the subjects' average percentage of correct answers according to the intervention type and stage. The ANOVA of repeated measures only showed a significant effect for the stages of the therapeutic process (F(5,40)=32.452, p<0.01). That is, it detected effects for the initial assessment of production (or pre-therapy), perception in the other, perception itself and production, but it did not show a significant effect for the type of intervention (i.e., CG and GG) or the interaction between intervention*stages of the therapeutic process. As shown in Figure 2, Scheffé's post hoc test revealed a statistical difference between pre-therapy and the other therapeutic intervention sessions and between the stages of perception (in the other and oneself) and production (target words and probe words) for both groups.

**Table 2:** Mean percentage of correct answers for the groups according to the intervention stages

**Type of Intervention**	**Initial Assessment**		**Worked Skills**					
	**Prod. P.A.**	**Prod. P.S.**	**Perception in the Other P.A.**	**Perception in the Other P.S.**	**Self-perception P.A.**	**Self-perception P.S.**	**Prod. P.A.**	**Prod. P.S.**
CG	55.3%	54.0%	73.6%	73.6,%	75.8%	76.9%	78.5%	74.0%
GG	57.3%	56.5%	71.6%	69.8%	70.4%	71.0%	62.5%	61.3%

**Caption:** P.A. = Target word; P.S. = Probing word; Prod. = Production

**Caption:** Pre-Therapy-Target = Pre-Therapy target words; Pre-Therapy-Sond. = Pre probing word therapy; Percept. - other = Perception in the other; Percep. - itself = Perception itself; Prod - Pal. Target = Production of target words; Prod - Pal. Sond = Production of probe words

**Figure 3:**
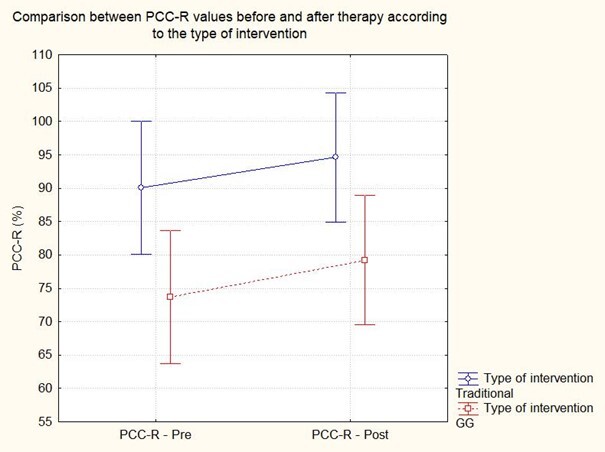
Comparison between PCC-R values before and after therapy according to the type of intervention

Table 3 presents the pre- and post-therapy PCC-R values of the subjects in both therapy groups and the number of sessions each subject needed to achieve the correct production of the target words and/or probe words with at least 85% correct. The ANOVA of Repeated Measures was used to compare the PCC-R values of the CG and GG groups pre- and post-therapy. There was a significant effect for pre- and post-therapy conditions (F(1,8)=39.31, p>0.00) and for the type of intervention (F(1,8)=7.08, p<0.00). However, there was no significant effect for the interaction between the pre-post*type of therapy (F(1,8)=0.39, p=0.54). Although PCC-R values increased for all subjects when comparing pre- and post-therapy conditions, CG subjects had higher PCC-R values than GG subjects (Figure 3).

**Table 3:** Pre- and post-intervention PCC-R values and number of correct production sessions for each subject

**Therapy Group**	**Subject**	**PCC-R Pre-Intervention**	**PCC-R Post-Intervention**	**# of sessions necessary to achieve a minimum of 85% of correct production**
**GG**	S2	87.1%	95.5%	3
	S3	77.6%	80.5%	10*
	S7	62.9%	72.2%	10*
	S8	83.5%	86.1%	10*
	S9	57.7%	61.7%	3
**CG**	S1	89.7%	93.2%	6
	S4	91.5%	95.6%	1
	S5	92.2%	97.1%	7
	S6	94.1%	97.0%	3
	S10	82.9%	90.2%	9

* Subject did not reach the minimum 85% correct production in 10 sessions

**Caption:** GG = Game Group; CG = Control Group; PCC-R = Percentage of Correct Consonants

**Caption:** PCC-R Pre = Percentage of Correct Consonants Pre-Therapy; PCC-R- Post = Percentage of Correct Consonants Post-Therapy

**Figure 4:**
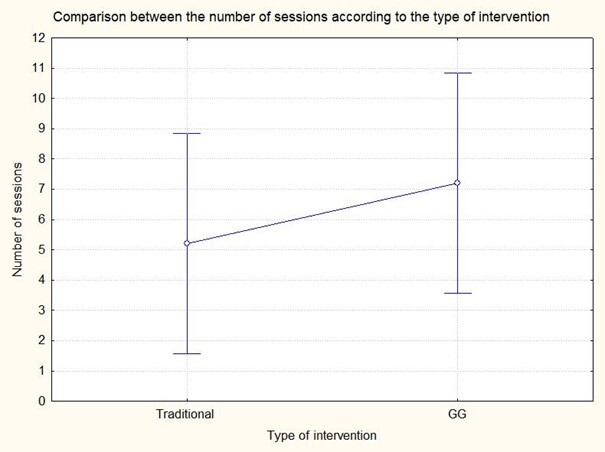
Comparison between the number of sessions according to the type of intervention

Regarding the comparison of the number of sessions necessary for the subjects to reach at least 85% of correct production of the worked target sounds (/ɾ/) or (/l/), there was no significant difference based on the One-Way ANOVA results (F(1,8)=0.80, p=0.39). Moreover, there was no difference between the number of sessions as a function of the type of therapy. These results are presented in Figure 4.

## DISCUSSION

The present study aimed to compare the effectiveness of phonological therapy associated with the gamification strategy and traditional therapy in subjects with PD. It was expected that the subjects submitted to the phonological intervention associated with the gamification strategies would present a better performance in terms of the percentage of correct answers of the worked skills and a shorter therapeutic time when compared to the subjects submitted to the traditional phonological therapy.

### Therapeutic efficacy of therapies was not associated with gamification strategies

Concerning group performance, we found no differences in accuracy or intervention stage (Figure 2) or the number of sessions (Figure 4), refuting the study's hypothesis. 

These results are consistent with previous studies^(14,15)^ that did not detect a statistically significant difference in speech production performance when evaluating interventions with and without gamification strategies. 

In a study mentioned^(14)^, the authors proposed that this result is due to the intervention consisting of only one weekly 30-minute session for eight weeks, a relatively short period to detect differences between the two interventions. In the present study, the consultations were also carried out for eight weeks but with two 50-minute sessions per week and did not yield significant differences.

On the other hand, in another study^(12)^ reported that a computer-based gamification strategy favors more changes in the subjects' phonological system when compared to traditional therapy. However, the authors of this study warned of the need to conduct further research with an expansion of the sample to confirm the findings, considering that only four subjects participated in the study. 

Additionally, another study showed a gamification strategy using the Speech Intervention Software (SIFALA), which allows subjects to explore and achieve treatment objectives^(21)^. The authors found that SIFALA improved correct speech sound production, facilitated lexical representation and augmented phonetic-phonological system information storage. Another study^(22)^ applied questionnaires aimed at speech therapists and individuals with PD to verify the usability and usefulness of the KeRa Puzzle digital game in therapeutic intervention. The findings showed that gamification had satisfactory usability and made the sessions more playful.

### Explanatory hypotheses for the non-difference in performance between the interventions

Factors such as familiarity with the computer, subject age, motivational aspects, family participation, number of subjects and intra-group heterogeneity could account for the lack of difference between the interventions.

Although the familiarity with the computer was not an analyzed variable, in the present study, three GG subjects (S2, S7 and S9) had knowledge and interest in operating the computer and electronic games. This fact favored engagement throughout the use of the tool in the sessions. In contrast, subjects S3 and S8 (also belonging to the GG) never used a computer and, initially, had difficulties handling the electronic device, making it challenging to engage and motivate with the proposed game immediately.

Concerning age, the GG subjects S2, S7 and S9, aged 5:11, 5:5 and 6:11, respectively, showed more interest in and sustained attention to the game than subjects S3 and S7, aged 4:11 and 5:0. A vital factor in younger subjects was the need to enhance gamification elements such as ranking, scoring and interactive awards during the activity to arouse their interest and encourage them to continue and to recognize their potential.

One of the elements of gamification is the motivational aspect^(1,2)^. It was observed that the effective combination of intrinsic (own and internal desire) and extrinsic (proposed gamification) motivations contributed to the level of motivation and engagement of the GG subjects. However, this aspect was the same compared to CG subjects.

Regarding family participation, previous studies have highlighted the relevance of family participation of parents/guardians in the intervention process to contribute to therapeutic efficacy^(23-25)^. This aspect was not considered in the analysis of the study. The family participation (i.e., performing the requested home activities during the therapeutic process) of the subjects participating in the present study was quite heterogeneous. This result could be an influential factor in the performance and therapeutic interest of the subjects, regardless of the type of intervention.

Concerning the number of subjects, our sample size did not allow for a generalization of the results. Due to this limitation, it is recommended that future randomized studies be carried out with more participants.

Furthermore, intra-group heterogeneity in the degree of PD severity within each group could account for the lack of difference between the interventions. In the CG (subjects S1, S4, S5, S6 and S10), most suppressed the liquid replacement processes at the end of the therapeutic process, except for S10, who presented 75% of correct production of the contrastive phoneme /ɾ/. When comparing subject S10 with the other subjects belonging to the same group, it was noted that he presented a higher degree of TF severity (slightly-moderate) and other phonological processes beyond the liquid class.

In contrast, in the GG, composed of subjects S2, S3, S7, S8 and S9, only S2 suppressed the worked phonological process. This subject was the only case that presented mild severity of PD and phonological processes only in the liquid class. The other group participants displayed higher PD severity and other phonological processes involving different classes.

### Performance differences between the skills worked in both intervention models

Although the main focus of the present study was not to compare the performance of the subjects in relation to the skills worked on in therapy, it was observed that in both groups, the pre-therapy conditions showed a low percentage of the correctness of the target sounds compared to the stages of perception (in the other and oneself) and speech production. This result means that from the beginning of the intervention process, all subjects already present a change in accuracy.

Among the stages, the accuracy (% of correct answers) and perception (in the other and oneself) differ from the accuracy of speech production in both groups. The subjects showed better performance in perception than in speech production. This result corroborates the assumption of a study^(26)^ exploring the possibility that the perception skill precedes the speech production skill. In other words, for an individual to appropriately produce a specific contrastive phoneme, they must perceive the phonetic properties- phonological and then substantiate these properties in their productions.

Another aspect to consider is that no univocal correlation exists between speech production and perception. As mentioned in a previous study^(27)^, the authors point out that the existing correlation between production and perception depends on the phonological class and that speech perception errors do not mirror speech production errors. Another study^(28)^ reported a significant correlation between speech production skills and perception of the subject's atypical speech production. This result suggests that assessing these skills seems to access the same underlying phonological representation. In this sense, if a subject has not established the underlying representation for a given phonological contrast, it will affect both skills: perception of the other's speech and perception of their speech, since the performance in perception skills requires access to a symbolic system, which may cause or contribute to deficits in speech production and perception. Therefore, considering the studies mentioned above, the speech perception ability directs us to an important implication in the rehabilitation process in phonological-based models since its inclusion in the evaluation and intervention could maximize therapeutic efficacy.

Regarding PCC-R, differences were observed when comparing pre- and post-therapy and group values (Figure 3). As previously mentioned, both groups' PCC-R values increased after therapeutic intervention, but the subjects of the CG group had higher PCC-R values than those of the GG. It should be noted that even before the therapeutic intervention (pre-therapy), the CG subjects already had higher PCC-R values than the GG. This fact highlights a considerable limitation of this study since the ideal would be to balance the distribution of subjects in each group according to the severity of the PD (i.e., PCC-R values).

## CONCLUSION

Both intervention models (traditional and gamification) improve the subject's phonological performance from the first session. There was no difference in therapy time or between the mean percentage of correct answers in the production of target words between the two approaches. A notable therapeutic implication is the possibility of using a computer-based gamification strategy to obtain results similar to those expected in traditional therapy.

The development of the present study aimed to contribute to the scientific discussions about therapy in the field of Clinical Phonology, favor the establishment of interventional processes of speech therapy mediation with gamification strategies and encourage the construction of new gamification strategies considering stages of perception and speech production.
