# Supplementary material for: Efficacy in the use of gamification strategy in phonological therapy
Source: Codas. 2023 Sep 8;35(6):e20220181. doi: 10.1590/2317-1782/20232022181en (PMC10547138; doi:10.1590/2317-1782/20232022181en)
Supplement: Translation

# Translation: Eficácia no uso de estratégia de gamificação na terapia fonológica

**DOI:** 10.1590/2317-1782/20232022181pt

**Authors:** Thalia Freitas da Silva, Grazielly Carolyne Fabbro Ribeiro, Cássio Eduardo Esperandino da Silva, Mayara Ferreira de Assis, Henrique Dezani, Larissa Cristina Berti

**Affiliations:** 1 Departamento Fonoaudiologia, Faculdade de Filosofia e Ciências, Universidade Estadual Paulista “Júlio de Mesquita Filho” - UNESP - Marília (SP), Brasil.; 2 Departamento Ciência da Computação, Faculdade de Tecnologia - FATEC - São José do Rio Preto (SP), Brasil.

**Keywords:** Transtorno Fonológico, Fonoterapia, Percepção da Fala, Gamificação, Terapia Assistida por Computador, Jogos Experimentais

## Abstract

**Objetivo:**

comparar as eficácias da terapia fonológica tradicional e terapia fonológica associada à estratégia de gamificação em sujeitos com Transtorno Fonológico (TF).

**Método:**

participaram dez sujeitos com TF que apresentavam o processo de substituição de líquidas. Os sujeitos foram randomizados em dois grupos: terapia fonológica tradicional (grupo controle - GC) e terapia fonológica associada a estratégia de gamificação mediada por computador (grupo gamificação - GG). A intervenção fonológica compreendeu, para ambos os grupos, etapas de percepção e produção de fala. As intervenções se diferenciaram na etapa de percepção, na qual o GG foi submetido ao jogo com estratégia de gamificação. Ao final de cada sessão, foram registrados a produção de fala dos sujeitos (% de acerto) para cada etapa terapêutica, a partir de palavras-alvo e palavras-sondagem. Para análise foram considerados: média de acerto dos sujeitos para cada etapa terapêutica; valores de PCC-R (Porcentagem de Consoantes Corretas Revisado) pré e pós terapia; além do número de sessões utilizadas para se atingir 85% de produção correta.

**Resultados:**

não houve diferença estatisticamente significante entre os tipos de intervenção considerando a média de acertos das produções e o número de sessões. Houve efeito significante para as condições pré e pós terapia na comparação dos valores de PCC-R para ambos os modelos. Os sujeitos do GC tiveram os valores de PCC-R maiores do que as do GG.

**Conclusão:**

ambos os modelos de intervenção apresentam resultados semelhantes, propiciando melhora no desempenho fonológico do sujeito desde a primeira sessão.

## INTRODUÇÃO

A gamificação, advinda do inglês *gamification*, refere-se ao uso de mecanismos de jogos, brinquedo, brincadeira ou ludicidade (analógicos ou digitais) orientados com o objetivo de despertar engajamento entre um público específico. Essas estratégias compõem recursos de game-design e elementos de jogos em contextos não-game, sendo o seu diferencial a capacidade de estabelecer recompensas, pontuação, desafios, reforços, *feedback* e *ranking* ^(1-2)^.

Dentre as áreas do conhecimento que podem se beneficiar de estratégias associadas à gamificação encontra-se a Fonoaudiologia; que pode fazer uso destas estratégias, tanto no processo de avaliação, quanto no processo de intervenção.

Nos últimos anos, em todas as áreas da Fonoaudiologia vem aumentando paulatinamente o uso de estratégias de gamificação, associadas ou não a jogos. Como consequência, observa-se como um produto concreto da utilização deste recurso a proposição e a comercialização de diversos *softwares* envolvendo jogos para avaliação e terapia de sujeitos com distintas alterações da comunicação, tais como: “Pedro em uma noite Assustadora”, cuja finalidade é estimular a consciência fonológica e fonoarticulatória; o Instrumento de Avaliação Fonológica (INFONO), voltado para facilitar o processo de avaliação de sujeitos com alterações de fala; além do “Auxiliar na Reabilitação de Distúrbios Auditivos (SARDA)”, que inclui a função de estimular as habilidades de processamento cognitivo-auditivo-visual^(3-6)^.

Particularmente, dentre os potenciais usuários desses materiais, encontram-se aqueles sujeitos que apresentam o diagnóstico de Transtorno dos Sons da Fala - especificamente Transtorno Fonológico (TF). Os sujeitos com TF apresentam produção de fala não esperada para a idade e estágio de desenvolvimento, ou seja, continuam a empregar regras de simplificação, designadas de processos fonológicos, para além da idade esperada sem que haja etiologia orgânica aparente^(7)^.

Ao longo dos anos, tem se intensificado estudos que englobam a intervenção fonológica com o intuito de averiguar sua eficácia e eficiência nessa população, uma vez que o processo de intervenção em sujeitos com TF tem como objetivo principal reorganizar o sistema fonológico alterado, de modo a atingir o sistema de um adulto típico para o Português Brasileiro melhorando, consequentemente, a inteligibilidade de fala^(8-11)^.

Para tanto, os sujeitos precisam estar motivados - o suficiente - para realizarem atividades propostas na terapia que envolva a habilidade em que eles têm maior dificuldade: a produção de fala.

Nesse sentido, torna-se um desafio diário para o fonoaudiólogo clínico pensar em estratégias que motivem os sujeitos com TF propiciando trabalhar as suas dificuldades de produção, tendo em vista que, em geral, o interesse do público infantil está voltado às diversas tecnologias, tal como o uso do computador^(12)^.

Constata-se que estratégias de gamificação com o uso do computador no âmbito da terapia fonológica podem ser ferramentas essenciais e complementares quando comparadas aos métodos tradicionais de terapia, ou seja, aqueles em que são propostas figuras, associadas ou não à jogos, para que o sujeito as nomeie.

No Brasil, uma pesquisa realizada com o uso do *software FonoSpeak* ^(12)^ - cujo objetivo é auxiliar na aquisição e treinamento de fonemas - em conjunto com atividades elaboradas no programa *Microsoft Office Power Point*, os autores verificaram que o grupo de terapia submetido a estratégias de gamificação com o recurso do computador obteve maior número de produções corretas quando comparado ao grupo de sujeitos submetidos à terapia tradicional, ou seja, sem uso de estratégias de gamificação.

Em concordância com o estudo citado acima, uma pesquisa^(13)^ com objetivo de avaliar a eficácia de programas terapêuticos com o uso do computador em sujeitos com Distúrbio dos Sons da Fala, incluindo o TF, a partir da revisão de literatura de 14 estudos, verificou que embora a base de evidências com o uso do computador ainda esteja ganhando espaço, de modo geral, a literatura mostrou que esse recurso pode ser um complemento útil para a terapia.

Todavia, há evidências contraditórias na literatura sobre o benefício do uso de estratégias de gamificação com o recurso do computador na terapia de sujeitos com TF. Neste estudo^(14)^, particularmente, foi comparado o desempenho de sujeitos com TF a partir da divisão de três grupos: sujeitos submetidos à terapia com o uso de estratégias de gamificação (*software* experimental com jogos de computador interativos); sujeitos submetidos à terapia tradicional; e sujeitos que não foram submetidos a qualquer terapia. A partir da análise do desempenho de produção de fala dos sujeitos, não houve diferença significativa entre nenhum dos três grupos.

Semelhantemente, em outro estudo^(15)^ foi feita a comparação da eficácia da intervenção fonológica tradicional com a intervenção fonológica baseada no uso do *tablet*, sendo assim, os sujeitos foram randomizados para cada grupo. Os autores concluíram que não houve diferença estatística entre os grupos, isso significa que independente do grupo, os sujeitos obtiveram porcentagens de produções de fala corretas na condição pós-terapia.

Há ainda na literatura nacional, uma pesquisa^(16)^ distinta das supracitadas, que buscou descrever a frequência da ferramenta do *tablet* durante intervenção de sujeitos com TF. Os jogos de gamificação utilizados no *tablet* não eram de cunho fonoaudiológico, mas sim adaptados ao trabalho em tarefas de imitação/nomeação aos níveis de sílabas, palavras e frases. O *tablet*, era utilizado como uma via auxiliar, sendo assim, ficava disponível para que o sujeito solicitasse ou não sua utilização durante a terapia. No estudo citado, pertenceu quatro sujeitos com faixas etárias entre cinco anos e cinco meses a onze meses e, como resultado, apontou que todos os sujeitos solicitaram o *tablet* com frequência significativa no decorrer das sessões, funcionando como recurso motivador, contudo, não decisivo para evolução dos casos.

Os estudos, tomados juntos, mostram não apenas resultados divergentes sobre a vantagem do uso de estratégias de gamificação na terapia fonológica, como também mostram baixa evidência científica, na medida em que a amostragem não foi aleatória ou o método utilizado não permitiu a comparação entre propostas terapêuticas distintas.

Assumindo que o uso de estratégias de gamificação no processo terapêutico de sujeitos com TF poderia favorecer o engajamento dos sujeitos nas atividades e, consequentemente, favorecer o aprendizado da habilidade de produção de fala trabalhado na terapia; hipotetizou-se que sujeitos que recebem intervenção fonológica associada às estratégias de gamificação apresentariam melhor desempenho em termos de porcentagem de acerto da habilidade trabalhada e um menor tempo terapêutico, quando comparadas com os sujeitos submetidos à terapia fonológica tradicional.

Portanto, este estudo objetivou comparar a eficácia da terapia fonológica associada à estratégia de gamificação com a eficácia da terapia tradicional em sujeitos com TF.

## MÉTODO

Trata-se de um estudo longitudinal prospectivo. Este estudo foi aprovado pelo Comitê de Ética em Pesquisa (CEP) da Faculdade de Filosofia e Ciências/Universidade Estadual Paulista (FFC/UNESP), Campus Marília, sob o protocolo nº 4.615.118.

Os pais e/ou responsáveis pelos sujeitos assinaram o Termo de Consentimento Livre e Esclarecido, autorizando a participação dos mesmos no projeto, bem como a publicação dos resultados. Esclarece-se, ainda, que a cada início das sessões de intervenção terapêutica, era perguntado para os sujeitos participantes se eles aceitariam participar da sessão. Portanto, todos os sujeitos expressaram o seus respectivos aceites.

Participaram do estudo dez sujeitos, seis do gênero masculino e quatro do feminino na faixa etária entre quatro anos e onze meses a sete anos e três meses de idade, com diagnóstico de TF e que apresentavam o processo fonológico de substituição de líquida não-lateral por lateral ou vice-versa (/ɾ/ → [l] ou /l/ → [ɾ]).

A amostra recrutada totalizou-se em 86 sujeitos a partir de triagem fonoaudiológica, que incluiu avaliação de fala e de linguagem, na Unidade Básica de Saúde e no Centro Especializado em Reabilitação (CER II). Ambos são localizados no interior do Estado de São Paulo, na cidade de Marília, portanto, o processo de amostragem do presente estudo foi por conveniência.

Os critérios de inclusão da pesquisa foram ter idade entre quatro a oito anos; apresentar diagnóstico de TF - com a presença do processo de substituição de líquida não-lateral por lateral ou vice-versa, independente da gravidade do TF e de outros processos fonológicos associados; pais e/ou responsáveis demonstrarem interesse e disponibilidade para participar do programa de intervenção terapêutico proposto.

Os critérios de exclusão foram alteração estrutural significativa dos órgãos fonoarticulatórios; presença de comorbidades além da queixa de produção de fala; queixa e/ou alterações auditivas e a não adesão ao programa de intervenção proposto ou possíveis desistências.

Para estabelecer o diagnóstico de TF e caracterizar os processos fonológicos, todos os sujeitos foram submetidos à avaliação fonológica com o “Instrumento de Avaliação de Fala para Análise Acústica” - IAFAC^(17)^. Esse instrumento é composto por 96 palavras que contemplam todos os fonemas consonantais do Português Brasileiro (PB) em contexto de /i a u/ nas posições acentuadas. Para essa avaliação, foram utilizadas apenas 28 palavras, em que todos os fonemas do PB ocorrem apenas no contexto da vogal /a/.

Ademais, foi realizado o cálculo do índice de gravidade do TF por meio da Porcentagem de Consoantes Corretas-Revisado (PCC-R) que se refere à porcentagem de consoantes produzidas corretamente em relação percentual ao total de consoantes contidas na amostra de fala obtida, além disso, para o cálculo deste índice são considerados como erros somente os fonemas substituídos ou omitidos^(18)^.

Para classificação dos diferentes graus do TF, foram considerados os critérios: a) leve: acima de 85% de acertos; b) levemente moderado: entre 65% e 85% de acertos; c) moderadamente severo: entre 50% e 65% de acertos; d) severo: abaixo de 50% de acertos^(19)^.

Os sujeitos também foram submetidos à avaliação audiológica para pesquisa de limiares auditivos para descarte de possíveis alterações.

Após a caracterização dos sujeitos, os mesmos foram submetidos a um sorteio e randomizados em dois grupos, sendo eles: terapia fonológica tradicional (grupo controle - GC) e terapia fonológica associada à estratégia de gamificação mediada por computador (grupo gamificação - GG). Ambos os grupos (GC e GG), pertenceram 5 sujeitos cada um, conforme descrito na Tabela 1, o perfil de cada sujeito.

**Tabela 1:** Perfil dos sujeitos pré-intervenção terapêutica

Sujeito	Sexo	Idade	Processo fonológico - substituição de Líquida	PCC-R Pré-intervenção	Gravidade do TF	Grupo
S1	M	6a 5m	L por N-L	89,7%	Leve	GC
S2	M	5a 11m	N-L por L	87,1%	Leve	GG
S3	F	4a 11m	N-L por L	77,6%	Levemente- moderado	GG
S4	F	7a 3m	N-L por L	91,5%	Leve	GC
S5	M	5a 11m	N-L por L	92,2%	Leve	GC
S6	M	5a 5m	N-L por L	94,1%	Leve	GC
S7	F	5a5m	N-L por L	62,9%	Levemente- moderado	GG
S8	M	5a0m	N-L por L	83,5%	Levemente- moderado	GG
S9	M	6a11m	N-L por L	57,3%	Moderadamente Severo	GG
S10	F	6a2m	N-L por L	82,9%	Levemente- moderado	GC

**Legenda:** M = Masculino; F = Feminino; L por N-L = Substituição de Líquida Lateral por Não-Lateral; N-L por L = Substituição de Líquida Não-lateral por Lateral; GG = Grupo Gamificação; GC = Grupo Controle

No programa de intervenção de base fonológica realizado para os grupos GC e GG, totalizaram-se 16 sessões de atendimentos fonoaudiológicos individuais, com frequência de duas vezes por semana e duração de 50 minutos cada -, no contraturno escolar dos sujeitos.

Para a realização da intervenção, foram selecionadas 30 palavras-alvo com os fonemas /l/ e /ɾ/ em critério de ISDP (Início de Sílaba Dentro de Palavra), trabalhadas durante as etapas de intervenção e outras 30 palavras-sondagem, isto é, palavras não trabalhadas em terapia que serviram para observar as generalizações realizadas pelos sujeitos. Além disso, parte das palavras selecionadas se referem à pares-mínimos, ou seja, palavras que se diferem em apenas um fonema, conforme ilustrado no Quadro 1.

**Quadro 1:** Palavras trabalhadas durante o processo de intervenção terapêutica nos grupos GC e GG

PALAVRAS-ALVO		PALAVRAS-SONDAGEM	
/ɾ/	**/l/**	**/ɾ/**	**/l/**
VERA	VELA	VARETA	VALETA
MARA	MALA	CARANGO	CALANGO
CARO	CALO	BARÃO	BALÃO
PURO	PULO	PÁRIO	PÁLIO
CARA	CALA	CARUSO	CALUSO
SARA	SALA	PERU	PELU
VIRA	VILA	GARI	GALI
VARA	VALA	PORO	PÓLO
CORADO	COLADO	RARA	RALA
CERA	SELA	GARO	GALO
MORA	MOLA	GERA	PALA
MARINHA	MALINHA	FARINHA	BALA
MIRA	MILA	JARARACA	COLA
SARADA	SALADA	MARACÁ	BELA
CORAGEM	COLAGEM	PERA	PISTOLA

As 16 sessões de intervenção fonoaudiológica destinadas ao GC e GG foram compostas por etapas de percepção e produção de fala, sendo elas: 1) pré-intervenção - coleta inicial/ apresentação e contextualização das gravuras correspondentes às palavras selecionadas; 2) explicação do processo fonológico - a partir do valor contrastivo das palavras-alvo, foi explicado para o sujeito, por meio de um protótipo de órgãos fonoarticulatórios, que existem sons que a língua bate devagar como é o caso do /l/ e existem sons que a língua bate rápido como na produção de /ɾ/; 3) percepção no outro - o sujeito realizou percepção auditiva-visual imediatamente após a produção de fala dos sons-alvo do outro (terapeuta); 4) percepção em si - a partir da própria produção dos sons-alvo, o sujeito realizou simultaneamente a propriocepção auditiva-visual. Nesta habilidade, não foi exigida a produção correta do som-alvo, o sujeito deveria realizar apenas sua propriocepção; 5) produção - o sujeito deveria produzir o som-alvo corretamente (a partir de pistas facilitadoras) em palavra isolada e na construção de frases; e 6) pós intervenção - coleta final.

As intervenções se diferenciaram na etapa 3, relativa à percepção no outro, na qual o GC contou exclusivamente com atividades lúdicas de material físico na 3 e nas demais etapas, enquanto o GG, somente nessa etapa, foi submetido ao jogo de gamificação “Ho-ho roubaram as palavras” mediado pelo computador. Sendo assim, os sujeitos desse grupo deveriam perceber os sons-alvo a partir da fala do outro por meio do game.

O jogo foi desenvolvido em parceria com a equipe de profissionais da Faculdade de Tecnologia (FATEC) de São José do Rio Preto, no interior do estado de São Paulo, coordenado pelo Dr. Henrique Dezani.

A plataforma elaborada tem seu uso para computadores, com disponibilidade online e gratuita. “Ho-ho roubaram as palavras”, tem como personagem principal um papai-noel, no qual o intuito do game é engajar o sujeito a encontrar as 30 palavras-alvo escondidas pelo cenário do jogo, conforme pode-se acessar através do link apresentado nas referências^(20)^. Na Figura 1 está disposta a interface do jogo.

**Figura 1:** Interface do jogo de gamificação “*Ho-ho roubaram as palavras*”

**Fonte**: Unity WebGL Player | Papai Noel Game^(20)^

A medida em que o sujeito encontra as palavras-alvos, são apresentados os estímulos auditivos correspondentes à cada figura, produzidas pelo outro, para que o sujeito identifique os sons-alvos.

### Análise de dados

Para ambos os tipos de intervenção, foi realizado o registro do desempenho de cada sujeito, por meio de gravações de produção de fala, calculado a partir da porcentagem de acerto de cada habilidade terapêutica trabalhada das 30 palavras-alvo e 30 palavras-sondagem. As gravações de produções de fala dos sujeitos foram realizadas apenas ao final de cada sessão para posteriormente serem feitas as análises perceptivo-auditivas.

As gravações de produções de fala, foram postas sob análise de, ao menos, dois juízes que realizaram o julgamento baseado em três critérios: (A) acerto de produção do som-alvo, (E) erro de produção do som-alvo ou (G) produção gradiente. Totalizou-se 960 gravações para cada sujeito e foi necessário a presença de um terceiro juiz para confirmar a análise de 281 gravações de todos os sujeitos da amostra, sendo 118 gravações para palavras-alvo e 163 gravações para palavras-sondagem.

Para cada etapa terapêutica (percepção no outro, percepção em si e produção) foram consideradas as médias de acerto, em termos de porcentagem para cada sujeito do GC e GG.

Os valores de PCC-R foram comparados nas condições pré e pós-terapia em ambos os grupos. Também foi feita a análise do número de sessões utilizadas para se atingir 85% de produção correta entre os tipos de intervenção. As 16 sessões propostas foram realizadas, independentemente se atingiram ou não a produção correta estabelecida.

Análises estatísticas descritiva e inferencial foram feitas com o uso do *software* STATISTICA (versão 7.0). Utilizou-se a ANOVA de Medidas Repetidas para a comparação entre os desempenhos dos grupos (GC e GG) nas etapas de intervenção do processo terapêutico e nos valores do PCC-R pré- e pós-terapia. O teste *Pos hoc* utilizado foi o de *Scheffé*. Para a comparação entre o número de sessões nos dois tipos de intervenção, utilizou-se a ANOVA *One-way.* Estabeleceu-se um valor de α>0,05.

## RESULTADOS

A Tabela 2 apresenta a porcentagem média de acerto dos sujeitos em função do tipo de intervenção e das etapas de intervenção.

**Tabela 2:** Porcentagem média de acertos dos grupos de acordo com as etapas de intervenção

**Tipo de Intervenção**	**Avaliação Inicial**		**Habilidades trabalhadas**					
	**Prod. P.A.**	**Prod. P.S.**	**Percepção no Outro P.A.**	**Percepção no Outro P.S.**	**Percepção em Si P.A.**	**Percepção em Si P.S.**	**Prod. P.A.**	**Prod. P.S.**
GC	55,3%	54%	73,6%	73,6,%	75,8%	76,9,5%	78,5%	74%
GG	57,3%	56,5%	71,6%	69,8%	70,4%	71%	62,5%	61,3%

**Legenda:** P.A. = Palavra-alvo; P.S. = Palavra-sondagem; Prod. = Produção

A Anova de medidas repetidas somente mostrou efeito significante para as etapas do processo terapêutico (F(5,40)=32,452, p<0,01), ou seja, avaliação inicial da produção (ou pré-terapia), percepção no outro, percepção em si e produção; mas não apresentou efeito significante nem para o tipo de intervenção (GC e GG) e nem para interação entre intervenção*etapas do processo terapêutico. O teste *Post Hoc* de *Scheffé* mostrou diferença estatística entre a pré-terapia e as demais sessões da intervenção terapêutica e, ainda, entre as etapas de percepção (no outro e em si) e a produção (palavras-alvo e palavras-sondagem) para ambos os grupos, conforme ilustrado na Figura 2.

**Figura 2:** Comparação relativa em função dos tipos de intervenção terapêutica: Tradicional e GG

**Legenda:** Pré-Terapia-Alvo = Pré-Terapia palavras-alvo; Pré-Terapia-Sond. = Pré-terapia de palavras-sondagem; Percep. - outro = Percepção no outro; Percep. - em si = Percepção em si; Prod - Pal. Alvo = Produção de palavras-alvo; Prod - Pal. Sond = Produção de palavras-sondagem

A Tabela 3, apresenta os valores do PCC-R pré e pós-terapia dos sujeitos em ambos os grupos de terapia, bem como o número de sessões que cada sujeito precisou para atingir a produção correta das palavras-alvo e/ou palavras-sondagem com pelo menos 85% de acerto.

**Tabela 3:** Valores do PCC-R pré e pós-intervenção e número de sessões de produção correta de cada sujeito

**Grupo de terapia**	**Sujeito**	**PCC-R Pré-intervenção**	**PCC-R Pós-intervenção**	**Nº de sessões necessárias para atingir no mínimo 85% de produção correta**
**GG**	S2	87,1%	95,5%	3
	S3	77,6%	80,5%	10*
	S7	62,9%	72,2%	10*
	S8	83,5%	86,1%	10*
	S9	57,7%	61,7%	3
**GC**	S1	89,7%	93,2%	6
	S4	91,5%	95,6%	1
	S5	92,2%	97,1%	7
	S6	94,1%	97%	3
	S10	82,9%	90,2%	9

* Sujeito não atingiu em 10 sessões o mínimo 85% de produção correta

**Legenda:** GG = Grupo Game; GC = Grupo Controle; PCC-R = Porcentagem de Consoantes Corretas

Na comparação entre os valores do PCC-R pré e pós-terapia do grupo de sujeitos submetidos à terapia tradicional (GC) e terapia mediada por estratégia de gamificação (GG), utilizou-se a ANOVA de Medidas Repetidas. Como resultados, houve efeito significante para as condições pré e pós-terapia (F(1,8)=39,31, p>0,00) e para o tipo de intervenção (F(1,8)=7,08, p<0,00), mas não houve efeito significante para a interação entre pré-pós*tipo de terapia (F(1,8)=0,39,p=0,54). Embora os valores de PCC-R aumentaram para todos os sujeitos na comparação das condições pré e pós-terapia, os sujeitos do GC tiveram os valores de PCC-R maiores do que os sujeitos do GG, tal como ilustra a Figura 3.

**Figura 3:** Comparação entre os valores de PCC-R pré e pós-terapia em função do tipo intervenção

**Legenda:** PCC-R Pré = Porcentagem de Consoantes Corretas Pré-Terapia; PCC-R- Pós = Porcentagem de Consoantes Corretas Pós-Terapia

Em relação à comparação do número de sessões necessárias para que os sujeitos atingissem pelo menos 85% de produção correta do sons-alvo trabalhados (/ɾ/) ou (/l/) a ANOVA *One-Way* não mostrou diferença significante (F(1,8)=0,80, p=0,39). Isto é, não houve diferença entre o número de sessões em função do tipo de terapia. A Figura 4, ilustra o resultado obtido.

**Figura 4:** Comparação entre o número de sessões em função do tipo de intervenção

## DISCUSSÃO

O presente estudo objetivou comparar as eficácias da terapia fonológica associada à estratégia de gamificação e a terapia tradicional em sujeitos com TF. Esperava-se que os sujeitos submetidos à intervenção fonológica associada às estratégias de gamificação apresentassem melhor desempenho em termos de porcentagem de acerto das habilidades trabalhadas e um menor tempo terapêutico, quando comparados aos sujeitos submetidos à terapia fonológica tradicional.

### Eficácia terapêutica em terapias associadas e não associadas às estratégias de gamificação

No tocante ao desempenho dos grupos, os achados obtidos apontaram que não houve diferença, tanto em relação à acurácia nas etapas de intervenção (Figura 2) quanto ao número de sessões (Figura 4), refutando a hipótese do estudo.

Esses resultados concordam com os estudos^(14,15)^ que não mostraram diferença estatisticamente significante em relação ao desempenho da produção de fala entre intervenções com e sem estratégias de gamificação.

Em um dos estudos mencionados^(14)^, o resultado apresentado pode ser explicado devido à realização de apenas um atendimento semanal com duração de 30 minutos, no período de 8 semanas de intervenção; fato que poderia ser um considerado um espaço curto de tempo para evidenciar diferenças entre os dois tipos de intervenção.

No presente estudo, os atendimentos também foram realizados em 8 semanas com dois atendimentos semanais com duração de 50 minutos, não sendo observada diferença entre os grupos quanto ao número de sessões.

Em contrapartida, esses achados divergem daqueles apresentados em outro estudo^(12)^, no qual verificou-se que a estratégia de gamificação com o uso do computador favorece mais mudanças no sistema fonológico dos sujeitos quando comparado com a terapia tradicional. Todavia, os autores deste estudo alertaram para a necessidade de realizar outras pesquisas com ampliação da casuística para confirmação dos achados, considerando que apenas quatro sujeitos participaram da pesquisa.

Outrossim, outros autores também concordaram em uma pesquisa com os benefícios dos jogos de gamificação em terapia^(21)^, como demonstrado com o uso *Software* de Intervenção para Fala (SIFALA), já que o mesmo permitiu explorar e alcançar os objetivos do tratamento de sujeitos com TF que incluíram ensinar a produção correta dos sons da fala, facilitar a representação lexical e o armazenamento das informações a respeito do sistema fonético-fonológico.

Demais pesquisadores^(22)^, realizaram a aplicação de questionários direcionados para fonoaudiólogos e sujeitos com TF para verificar a usabilidade e utilidade do jogo digital *KeRa Puzzle* em intervenção terapêutica, e, os achados mostraram que o jogo de gamificação possuiu usabilidade satisfatória, além de poder ser um recurso interessante para tornar as sessões mais lúdicas.

### Hipóteses explicativas para a não diferença de desempenho entre os tipos de intervenção

Fatores como familiaridade com o computador, idade do sujeito, aspectos motivacionais, participação familiar, número de sujeitos e heterogeneidade intra-grupo podem estar na base da explicação da não diferença entre os tipos de intervenção.

Embora a familiaridade com o computador não tenha sido considerada como uma variável a ser analisada, observou-se que três sujeitos do GG (S2, S7 e S9) já tinham conhecimento e interesse em manusear o computador e jogos eletrônicos, esse fato favoreceu o engajamento durante todo o uso da ferramenta nas sessões. Entretanto, os sujeitos S3 e S8 (também pertencentes ao GG) nunca tinham utilizado o computador e, inicialmente, apresentaram dificuldades com o manuseio do dispositivo eletrônico, o que dificultou o engajamento e a motivação com o game proposto de modo imediato.

Outra possível hipótese explicativa diz respeito à idade dos sujeitos do GG, pois observou-se que os sujeitos S2, S7 e S9 com idades de 5:11, 5:5 e 6:11, respectivamente, apresentaram maior interesse no game e atenção sustentada ao compará-los com os sujeitos S3 e S7 com idades de 4:11 e 5:0. Um fator importante nos sujeitos de idades menores, foi a necessidade de potencializar durante a atividade os elementos da gamificação, como *ranking*, pontuação e premiação interativa para despertar o interesse dos envolvidos, encorajando-os a continuar e respeitando suas potencialidades.

Considerando que um dos elementos da gamificação é o aspecto motivacional^(1,2)^, foi observado que a combinação efetiva das motivações intrínseca (desejo próprio e interno) e extrínseca (jogo de gamificação proposto) contribuíram para nível de motivação e engajamento dos sujeitos do GG. Entretanto, não foi observado diferença entre este aspecto no GC.

No que se refere à participação familiar, estudos em âmbito nacional e internacional têm destacado a relevância da participação familiar dos pais/responsáveis no processo de intervenção para contribuição da eficácia terapêutica^(23-25)^. Esse aspecto não foi um fator considerado na análise do estudo. Porém, evidentemente, a participação familiar dos sujeitos participantes da pesquisa, traduzida na realização de atividades domiciliares solicitadas durante o processo terapêutico, foi bastante heterogênea, podendo ser considerada um fator influente no desempenho e interesse terapêutico dos sujeitos independentemente do tipo de intervenção aplicada.

Em relação ao número de sujeitos, a limitação do estudo está em relação ao tamanho da amostra, o que não permite uma generalização dos resultados. Em razão disso, recomenda-se que sejam feitos estudos futuros randomizados e com amostra ampliada.

Destaca-se também a heterogeneidade, em termos de grau de severidade do TF, presente no interior de cada grupo. No GC composto pelos sujeitos S1, S4, S5, S6 e S10, no final do processo terapêutico, a maioria destes suprimiram os processos de substituição de líquidas, exceto o S10 que ainda apresentou 75% de produção correta do fone contrastivo /ɾ/. Ao comparar o sujeito S10 com os demais sujeitos pertencentes ao mesmo grupo, notou-se que este apresentou maior grau de severidade do TF (levemente-moderado) e outros processos fonológicos para além da classe das líquidas.

Diferentemente, no GG, composto pelos sujeitos S2, S3, S7, S8 e S9, apenas o S2 suprimiu o processo fonológico trabalhado, considerando que este foi o único que apresentou gravidade Leve do TF e processos fonológicos somente na classe das líquidas. Os demais participantes do grupo, por sua vez, apresentaram maior grau de severidade do TF e outros processos fonológicos os quais envolviam diferentes classes.

### Diferenças de desempenho entre as habilidades trabalhadas em ambos os modelos de intervenção

Embora o foco principal do presente estudo não tenha sido comparar o desempenho dos sujeitos em relação às habilidades trabalhadas em terapia, observou-se que nos dois grupos as condições pré-terapia mostraram uma baixa porcentagem de acerto dos sons-alvo comparativamente com as etapas de percepção (no outro e em si) e produção de fala. Isso significa dizer que a partir do início do processo de intervenção todos sujeitos já apresentam uma mudança em termos de acurácia.

Dentre as etapas, a acurácia (% de acerto) de percepção (no outro e em si) se diferenciam da acurácia da produção de fala para os dois grupos. Os sujeitos apresentaram melhores desempenhos de percepção do que de produção de fala. Este resultado corrobora a assunção de um estudo^(26)^ sobre a possibilidade de a habilidade de percepção preceder a habilidade de produção de fala, ou seja, para que um sujeito produza apropriadamente determinado fone contrastivo, é necessário que o mesmo perceba as propriedades fonéticas-fonológicas para depois fundamentar essas propriedades em suas próprias produções.

Outro aspecto a ser considerado é o de que não existe uma correlação unívoca entre produção e percepção de fala. Conforme mencionado em estudo anterior^(27)^, os autores ressaltam que a correlação existente entre produção e percepção é dependente da classe fonológica e, ainda, que os erros de percepção de fala não espelham os erros de produção de fala.

Outro estudo^(28)^, particularmente, refere que a presença de uma correlação significativa entre as habilidades de produção de fala e percepção da própria produção de fala atípica do sujeito sugere que a avaliação de tais habilidades parece acessar a mesma forma de representação fonológica subjacente. Ou seja, se um sujeito que não estabeleceu a representação subjacente para um determinado contraste fonológico afetará ambas as habilidades: percepção da fala do outro e percepção de sua própria fala, uma vez que o desempenho nas habilidades de percepção requer o acesso a um sistema simbólico, podendo causar ou contribuir para déficits na produção e percepção da fala.

Ao considerar os supracitados, portanto, a habilidade de percepção de fala nos direciona para uma importante implicação no processo de reabilitação nos modelos de base fonológica, visto que, sua inclusão na avaliação e intervenção é uma medida valiosa para se atingir a eficácia terapêutica.

Em relação à comparação dos valores do PCC-R, observou-se diferenças tanto em relação às condições pré versus pós, quanto em relação aos grupos (Figura 3). Como apresentado anteriormente, ambos os grupos modificaram os valores do PCC-R após intervenção terapêutica. O GC apresentou maiores valores de PCC-R comparativamente ao GG.

No entanto, cabe destacar que mesmo antes da intervenção terapêutica (pré-terapia) os sujeitos do GC já apresentavam valores de PCC-R superiores do que o GG. Tal fato ressalta uma limitação considerável deste estudo, posto que, o ideal seria equilibrar a distribuição dos sujeitos em cada grupo de acordo com a gravidade do TF (valores de PCC-R).

## CONCLUSÃO

Ambos os modelos de intervenção (tradicional e gamificação) propiciam melhora no desempenho fonológico do sujeito a partir da primeira sessão. Não houve diferença no tempo de terapia nem entre a média da porcentagem de acerto na produção das palavras-alvo entre as duas abordagens.

Uma importante implicação terapêutica refere-se à possibilidade do uso de estratégia de gamificação com o uso do computador, podendo se obter resultados semelhantes àqueles esperados na terapia tradicional.

O desenvolvimento do presente estudo visou contribuir com as discussões científicas acerca da terapia no campo da Fonologia Clínica, favorecer com o estabelecimento de processos interventivos de mediação fonoaudiológica com estratégias de gamificação e incentivar a construção de novos jogos de gamificação que envolvam etapas de percepção e produção de fala.
